# Disturbance of skin sensation and autism spectrum disorder: A bidirectional Mendelian randomization study

**DOI:** 10.1002/brb3.3238

**Published:** 2023-09-05

**Authors:** Xiao Zhong, Letong Wang, Lin Xu, Jie Lian, Jie Chen, Xinxin Gong, Yongcong Shao

**Affiliations:** ^1^ School of Psychology Beijing Sport University Beijing China

**Keywords:** autism spectrum disorder, disturbance of skin sensation, Mendelian randomization

## Abstract

**Background and aim:**

Patients with autism spectrum disorder (ASD) commonly experience aberrant skin sensation sensitivity; however, the causal relationship is not yet clear. This study uses a bidirectional Mendelian randomization (MR) method to explore the relationship between disturbance of skin sensation (DSS) and ASD.

**Methods:**

Single‐nucleotide polymorphisms (SNPs) extracted from the summary data of genome‐wide association studies were used as genetic instruments. MR was performed using the inverse‐variance‐weighted method, with alternate methods (e.g., weighted median, MR‐Egger, simple mode, weighted mode, and MR‐pleiotropy residual sum and outlier) and multiple sensitivity analyses to assess horizontal pleiotropy and remove outliers.

**Results:**

The results of the analysis using six SNPs as genetic instruments showed that the DSS is associated with an increased risk of ASD (odds ratio = 1.126, 95% confidence interval = 1.029–1.132; *p* = .010). The results of the sensitivity analyses were robust with no evidence of pleiotropy. The reverse MR analyses showed no causal effects of ASD on DSS.

**Conclusion:**

This study's findings suggest that DSS has potential causal effects on ASD, whereas ASD has no effect on DSS. Thus, skin sensitivity may represent a behavioral marker of ASD, by which some populations could be subtyped in the future.

## Graphical abstract

Patients with autism spectrum disorder (ASD) commonly experience aberrant skin sensation sensitivity; however, the causal relationship is not yet clear. We used a bidirectional Mendelian randomization (MR) study to explore the causal relationship. Our innovative a bidirectional MR study found that the disturbance of skin sensation (DSS) has potential causal effects on ASD, whereas ASD has no effect on DSS. Thus, skin sensitivity may represent a behavioral marker of ASD, by which some populations could be subtyped in the future.

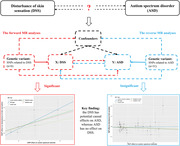

## Lay Summary

This study examines whether disturbance of skin sensation (DSS) has potential causal effects on autism spectrum disorder (ASD) using the bidirectional Mendelian randomization method. We believe our study makes a significant contribution to the literature because it provides more evidence of the causal effect of skin sensory abnormalities, such as touch, temperature, and tactile sensation, in ASD. Additionally, the findings are a reminder that the discovery of DSS as a biomarker in some ASD patients provides an objective and quantifiable indicator of the clinically relevant course of ASD, which can help to identify targets for intervention or treatment.

## 1 INTRODUCTION

Autism spectrum disorder (ASD) is a neurodevelopmental disorder that occurs in early childhood and has core symptoms of impaired social interaction and communication, a narrow range of interests, and stereotyped or abnormal behavior (Fung & Hardan, 2014; Volkmar & McPartland, 2014). The prevalence and diagnosis of ASD have increased dramatically in the past two decades, covering 1%–2.5% of the population. Autism has evolved into a major global public health problem (Lord et al., 2018); however, owing to the complexity of ASD symptoms (Frith, 2021), the pathogenesis of this disorder and its interventions remain unclear.

Previous studies have shown sensory abnormalities in children with ASD, who can exhibit varying degrees of under‐ or over‐responses (Ben‐Sasson et al., 2009). Furthermore, unlike previous versions of the DSM, the DSM‐5 includes sensory abnormalities (including hyper‐ or hyposensitivity) and an obsession with certain sensory stimuli to the stereotypical behavior category as diagnostic criteria for ASD (American Psychiatric Association, DSM‐5 Task Force, 2013). However, some controversies remain in this aspect. For example, the sense of touch, temperature, and pain in the skin, which is an important sensory organ, may be potential causes of the core symptoms of ASD, rather than simply symptoms themselves (Orefice et al., 2016; Yu et al., 2022).

Several studies have found that people with ASD often exhibit abnormal somatic or cutaneous sensations (Asmika et al., 2018; Ben‐Sasson et al., 2009), and many observational studies have shown patients with ASD have difficulties in atypical pain perception, expression, and pain assessment (Bogdanova et al., 2022; Moore, 2015; Ruelle‐Le Glaunec et al., 2021; Vaughan et al., 2020). However, skin is the most widely distributed and earliest developing sensory organ in the human body. It is an essential channel for humans to perceive information about their external environment (Lagercrantz & Changeux, 2009) and transmit physical characteristics and emotional information to the brain (Stilla & Sathian, 2008; Yu et al., 2022). Thus, some researchers have focused on the question of whether various skin sensations cause changes in the core symptoms of ASD (Robertson & Baron‐Cohen, 2017; Yu et al., 2022), which has led to the discovery of definitive new evidence. For instance, Orefice et al. (2019, 2016) found that early abnormal tactile responses may contribute to social impairment and anxiety‐like behaviors in ASD, and results by Huzard et al. (2022) suggested that early developmental petting promotes socially interactive behaviors and positive emotional experiences in adult mice.

Although some evidence now suggests that disturbance of skin sensation (DSS) may be one potential cause of the onset and progression of the core symptoms of ASD, relevant findings remain insufficient and lack reproducibility (Parellada et al., 2023). Therefore, more causal evidence is needed. Investigators have traditionally conducted randomized clinical trials to explore causal relationships, but the effect of confounding factors and ethical considerations can limit the usefulness of this approach (Allman et al., 2018). To some extent, bidirectional Mendelian randomization (MR) studies can partly address these questions.

MR is a data analysis technique used to assess etiologic inferences in epidemiologic studies. In many cases where randomized controlled trials are not possible, it allows genetic variation to be used as an instrumental variable (IV) in nonexperimental data to estimate the relationship between the exposure factor and outcome of interest, potentially providing information on causality (Burgess et al., 2013). The term “exposure factor” refers to a putative causal risk factor, also known as an intermediate phenotype, which can be a biomarker, a physical measurement, or any risk factor that may affect resolution. Generally, diseases are included as outcomes; however, these outcomes are not limited to a specific disease. MR theory can be established because MR utilizes the fixed nature of genes and Mendel's first and second laws of inheritance, that is, when meiotic gametes are formed, the parents’ alleles are randomly assigned to the offspring, and the relationship between genes and outcomes is not disturbed by common confounding factors such as postnatal environment, socioeconomic background, and behavioral habits, and the resulting causal time series are plausible (Zheng et al., 2017). Additionally, two main types of MR studies can be conducted: single‐sample randomization and two‐sample randomization. The first type requires measurements of the gene variant, risk factor, and health outcome from the same sample of participants, whereas the second requires two different study populations. In the two‐sample MR method, the gene variants and health outcomes are measured in one group, whereas the gene variants and risk factors are measured in another (Pierce & Burgess, 2013). The main advantage of two‐sample MR is that it does not produce false positive results when no sample overlaps (Burgess et al., 2020). The bidirectional aspect is the swapping of the roles of the exposure factor and outcome variable based on the two‐sample MR study, which can verify the existence of reverse causal effects.

In the current study, we aimed to investigate whether DSS has causal effects on ASD using a two‐sample MR method. First, DSS was used as an exposure factor, single‐nucleotide polymorphisms (SNPs) significantly associated with DSS were used as IVs, and ASD was used as an outcome variable to determine if DSS has a potential causal effect on ASD. Second, to exclude the possibility that ASD is causally associated with DSS outcomes, the MR analysis was conducted in the reverse direction (i.e., ASD as the exposure factor and DSS as the outcome variable). Based on the assumption that the skin is an important channel for information transmission (Yu et al., 2022), we hypothesized that a causal effect of DSS on ASD would hold, whereas a causal effect of ASD on DSS would not.

## 2 METHOD

### 2.1 Study design

**FIGURE 1:**
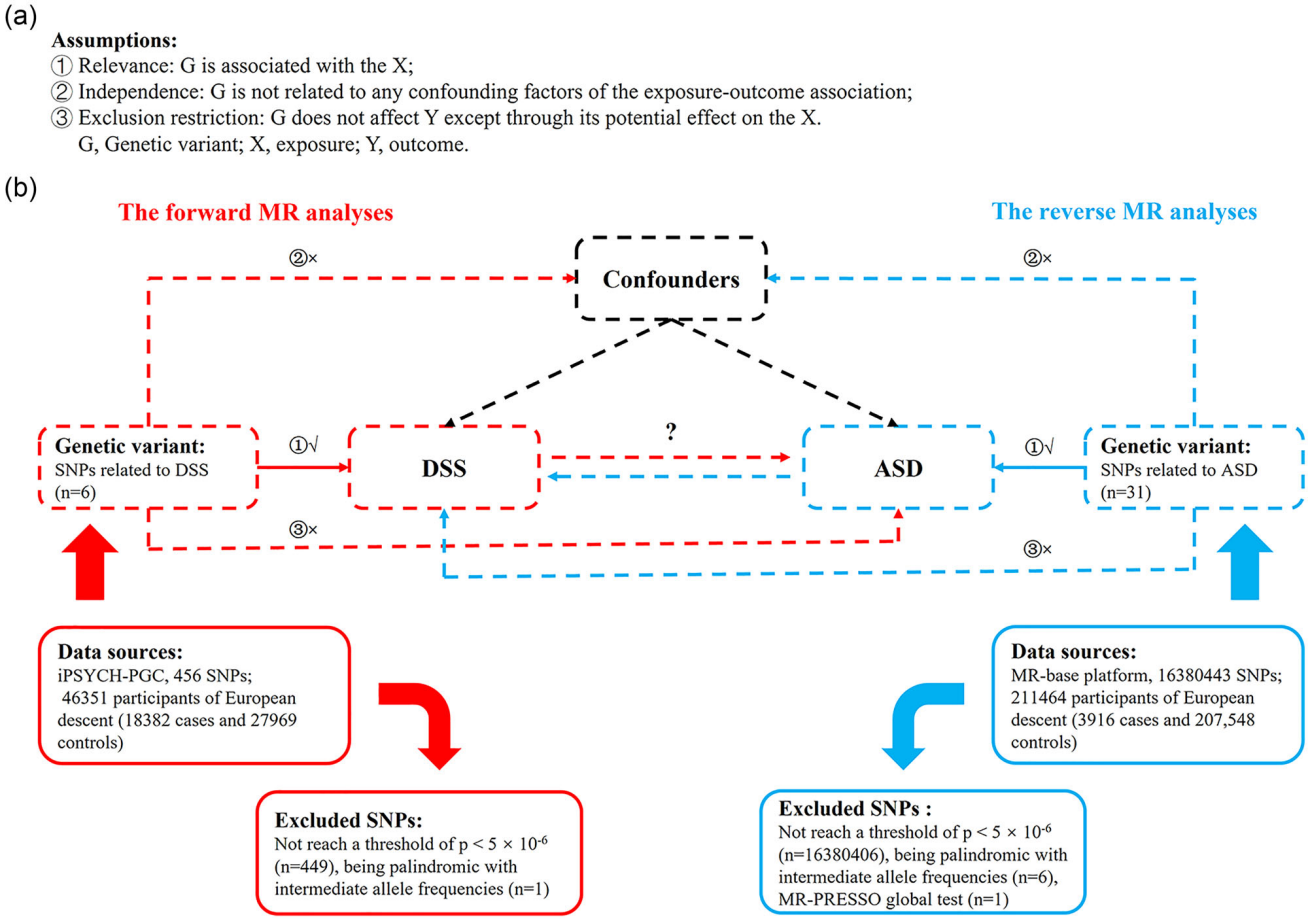
Description of the design used in this bidirectional Mendelian randomization (MR) study. (a) MR analyses dependent on three core assumptions. (b) Sketch of the study design. The red represents the forward MR analyses, with disturbance of skin sensation (DSS) as the exposure factor and autism spectrum disorder (ASD) as the outcome. The blue represents the reverse MR analyses, with ASD as the exposure factor and DSS as the outcome. SNPs, single‐nucleotide polymorphisms. *Source*: (b) Referenced from Huang et al. (2022).

A bidirectional MR study, based on a two‐sample MR method, was designed to estimate the causal relationship between DSS and ASD (see Figure 1). The MR analysis was first performed in one direction to determine the causal effect of DSS on ASD and then in the opposite direction. All the analyses were performed using summary‐level data from publicly available genome‐wide association studies (GWAS; https://gwas.mrcieu.ac.uk/). All data used are already in the public domain; therefore, no additional ethical approval was required.

### 2.2 Data source

#### 2.2.1 Data for autism spectrum disorder

We obtained information on the genetic associations with ASD from the GWAS collected by the Lundbeck Foundation Initiative for Integrative Psychiatric Research and the Psychiatric Genomics Consortium (iPSYCH‐PGC; Grove et al., 2019). The iPSYCH‐PGC has conducted the most comprehensive GWAS on ASD, including 46,351 participants (18,382 cases and 27,969 controls) selected from Europe. Of these, the iPSYCH ASD sample is a population‐based case‐cohort sample extracted from a baseline cohort comprising all children born in Denmark between May 1, 1981 and December 31, 2005 (Grove et al., 2019). Eligible children were singletons born to a known mother who was a resident of Denmark on their child's 1‐year birthday. The ASD register sample in the iPSYCH ASD GWAS includes 17,763 children (2820 female; 1873 IQ < 70), of which 3310 have been diagnosed with childhood autism, 1607 with atypical autism, 4622 with Asperger syndrome, 2042 with other pervasive developmental disorders (DDs), and 3753 with unspecified pervasive DDs.

#### 2.2.2 Data for disturbance of skin sensation

Summary data on ASD were obtained using the MR‐base platform (http://www.mrbase.org/). The study's 211,464 participants comprised 3916 cases and 207,548 controls with ASD. All the participants were of European ancestry.

### 2.3 Instrument selection criteria

The genetic instruments for ASD and DSS were extracted using the same criteria. We selected all the relevant SNPs at the genome‐wide significance (*p* < 5 × 10^−6^) threshold from each GWAS, and all were independent, that is a pairwise linkage disequilibrium (*r* ^2^ < .001 and distance >10 000 kb). *F*‐Statistics were used to evaluate the strength of the selected instruments (Burgess et al., 2016). Typically, an *F*‐statistic >10 is considered sufficiently informative for MR analyses (Palmer et al., 2012) and is calculated as follows:

(1) $$\begin{array}{c} F\;=\;\frac{R^{2}\times \left(N-2\right)}{1-R^{2}} \end{array}$$

where *R* ^2^ is the variance in exposure factors explained by a single specific SNP, and *N* is the sample size of the GWAS in the outcome variable.

### 2.4 Statistical analysis

The MR analyses utilized the random‐effects inverse‐variance‐weighted (IVW) method as the primary method to estimate potential bidirectional causal associations between DSS and ASD and its components. It provides a robust causal estimate in the absence of directional pleiotropy. This analysis was performed with data obtained, from the GWAS summary statistics, for the *J* genetic variants *𝐺_j_* (*j =* 1, …, *J*), effect values ($\hat{\beta }$ *_X𝑗_* and $\hat{\beta }$_𝑌𝑗_) for the corresponding exposures versus outcomes, as well as standard errors (se($\hat{\beta }$ *_X_*_𝑗_) and se($\hat{\beta }$_𝑌𝑗_)). The causal effect $\hat{\theta }$ *_j_* of exposure *X* on outcome *Y* can be obtained from a single genetic variant *𝐺_j_* using the Wald ratio method, which was calculated as follows:

(2) $$\begin{array}{c} \;{\hat{\theta }}_{j}=\frac{{\hat{\beta }}_{Yj}}{{\hat{\beta }}_{Xj}}\; \end{array}$$

its variance $\nu_{j}$ is obtained using the delta method, which was calculated as follows:

(3) $$\begin{array}{c} \;\nu_{j}=\frac{se{\left({\hat{\beta }}_{Yj}\right)}^{2}}{{\hat{\beta }}_{Xj}}\; \end{array}$$

A weighted summation of the effects of exposure on outcome, represented by the genetic tool consisting of *J* genetic variants, was obtained as follows:

(4) $$\begin{array}{c} \;{\hat{\theta }}_{IVW}=\frac{\sum_{j=1}^{J}w_{j}{\hat{\theta }}_{j}}{\sum_{j=1}^{J}w_{j}}\; \end{array}$$

where 𝑤*_j_* denotes the reciprocal of 𝑣*_j_*.

Its variance ($\hat{\theta }$ *_IVM_*) was obtained as follows:

(5) $$\begin{array}{c} v\;\left({\hat{\theta }}_{IVM}\right)=\frac{1}{\sum_{j=1}^{J}w_{j}}\; \end{array}$$

Furthermore, we used the weighted median, simple mode, weight mode, and MR‐Egger methods for alternative analyses. The weighted median and weighted mode methods allow for robust estimation in the presence of null IVs and MR‐Egger allows for robust estimation in the presence of horizontal multinomials (Zheng et al., 2017). For binary exposures, the causal estimate was presented as an odds ratio (OR) and a 95% confidence interval (CI) per log‐odds increment in the genetically determined risk of exposure.

We then performed tests for directional horizontal pleiotropy using the MR‐Egger intercept (Verbanck et al., 2018). We also tested the heterogeneity of the MR‐Egger regression and the IVW method using Cochran's *Q* statistics and funnel plots (Bae & Lee, 2019). Additionally, a sensitivity analysis was conducted by applying a leave‐one‐out analysis, which refers to the sequential elimination of one SNP locus and the use of the remaining SNP loci in MR analyses to test for the presence of bias caused by a particular SNP locus.

All the statistical analyses were performed using DevTools, TwoSampleMR, and MR‐PRESSO packages in R (version 3.6.3, www.r‐project.org/). All *p* values were two‐tailed. We adopted a *p* value <.05 to determine statistical significance in the MR analysis (Bonferroni corrected). A *p* value less than .05 was considered significant in the MR‐Egger test and heterogeneity test. All the data were retained to three decimal places.

## 3 RESULTS

### 3.1 The causal effect of DSS on ASD

Using seven SNPs as genetic instruments, we conducted an IVW MR analysis. One SNP (rs10190633) was removed for being palindromic with intermediate allele frequencies. Finally, six independent SNPs were used as genetic IVs for DSS. All *F*‐statistic values of the individual SNPs were greater than 10, ranging from 21.553 to 23.662, with an average of 22.584. Detailed information on the IVs for DSS is provided in Supporting Information Appendix 1.

**FIGURE 2:**
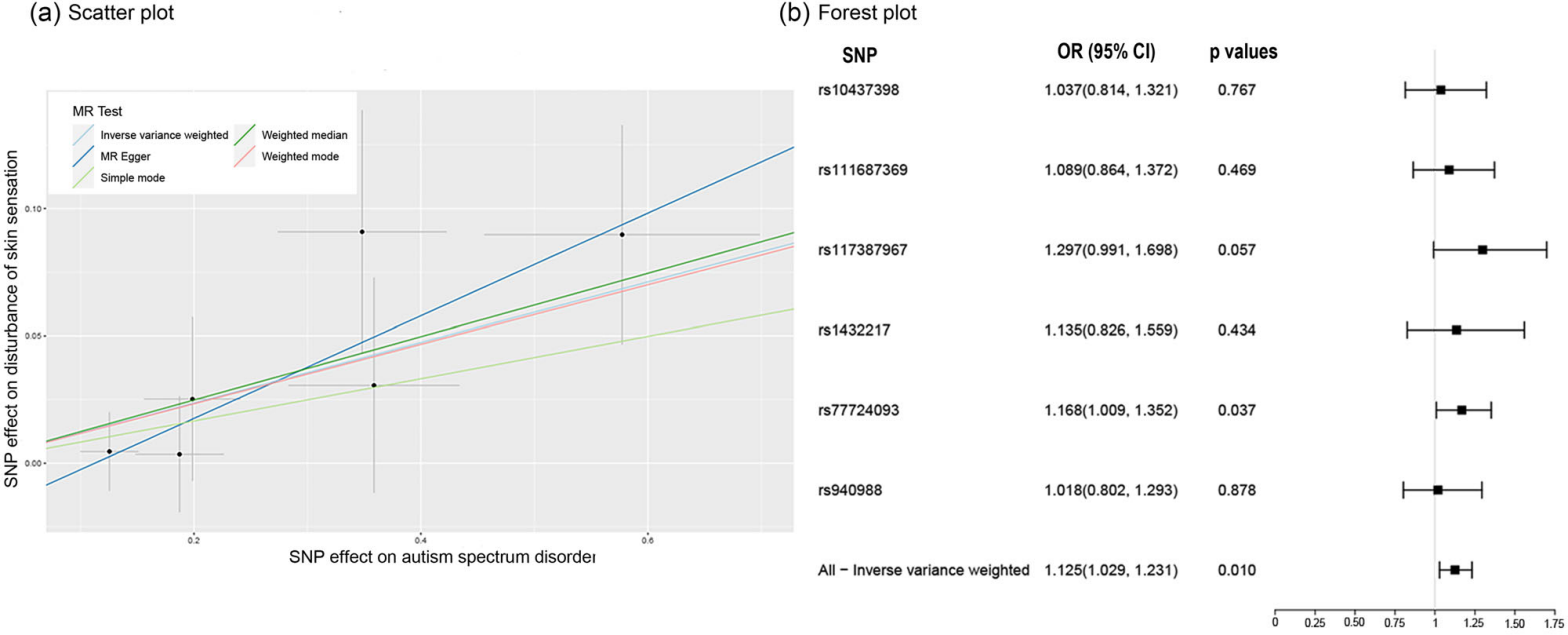
Mendel randomization diagram of causal relationship between disturbance of skin sensation (DSS) and autism spectrum disorder (ASD). (a) Scatterplot of single‐nucleotide polymorphism (SNP) potential effects on DSS versus ASD with the slope of each line corresponding to estimated Mendelian randomization (MR) effect per method. (b) Forest plot of individual and combined SNP MR‐estimated effects sizes. Data are expressed as raw odds ratio (OR) values with 95% confidence interval (CI).

The results of the MR analyses are shown in Figure 2 that genetically predicted DSS is associated with higher odds of ASD in the main IVW analyses, and the potential causal effect of DSS on ASD is significant (*OR* = 1.126, 95% CI = [1.029, 1.132]; *p* = .010). Similar results were obtained in the weighted median analyses (*OR* = 1.132, 95% CI = [1.025, 1.146]; *p* = .044; see Figure 2 and Table 1). The results were consistent across sensitivity analyses (MR‐Egger: Cochran's *Q* = 1.364, *p* = .850; IVW: Cochran's *Q* = 2.511, *p* = .775; see Supporting Information Appendices 2 and 3), none of the MR‐Egger intercepts significantly deviated from zero (MR‐Egger intercept = −.023, *p* = .344; see Supporting Information Appendix 4), and the MR‐pleiotropy residual sum and outlier (MR‐PRESSO) global tests were all statistically insignificant (*p* = .818; see Supporting Information Appendix 5).

**TABLE 1:** Mendelian randomization (MR) results for the relationship among disturbance of skin sensation and autism spectrum disorder.

Exposure	Outcomes	Method	nSNP	OR	or_lci95	or_uci95	*p*
Disturbance of skin sensation	Autism spectrum disorder	MR–Egger	6	1.223	1.025	1.459	.089
		Weighted median	6	1.132	1.003	1.278	.044
		IVW	6	1.126	1.029	1.232	.010
		Simple mode	6	1.087	0.919	1.284	.375
		Weighted mode	6	1.124	0.956	1.321	.217
Autism spectrum disorder	Disturbance of skin sensation	MR‐Egger	30	1.034	0.694	1.540	.432
		Weighted median	30	0.903	0.755	1.080	.077
		IVW	30	0.933	0.819	1.063	.061
		Simple mode	30	0.934	0.650	1.342	.294
		Weighted mode	30	0.915	0.661	1.267	.236

Abbreviations: IVW, inverse‐variance weighted; OR, odds ratio.

### 3.2 The causal effect of ASD on DSS

Using 37 SNPs as genetic instruments, we removed palindromic SNPs with intermediate allele frequencies. Next, 31 independent SNPs were used as genetic IVs for DSS. Then, because the MR‐PRESSO global tests were edge significant (.059), we removed another SNP (rs4916723; see Supporting Information Appendix 1). All *F*‐statistic values of the individual SNPs were all more than 10, ranging from 20.755 to 35.773, with an average of 24.179. Supporting Information Appendix 1 provides detailed information on ASD's IVs.

**FIGURE 3:**
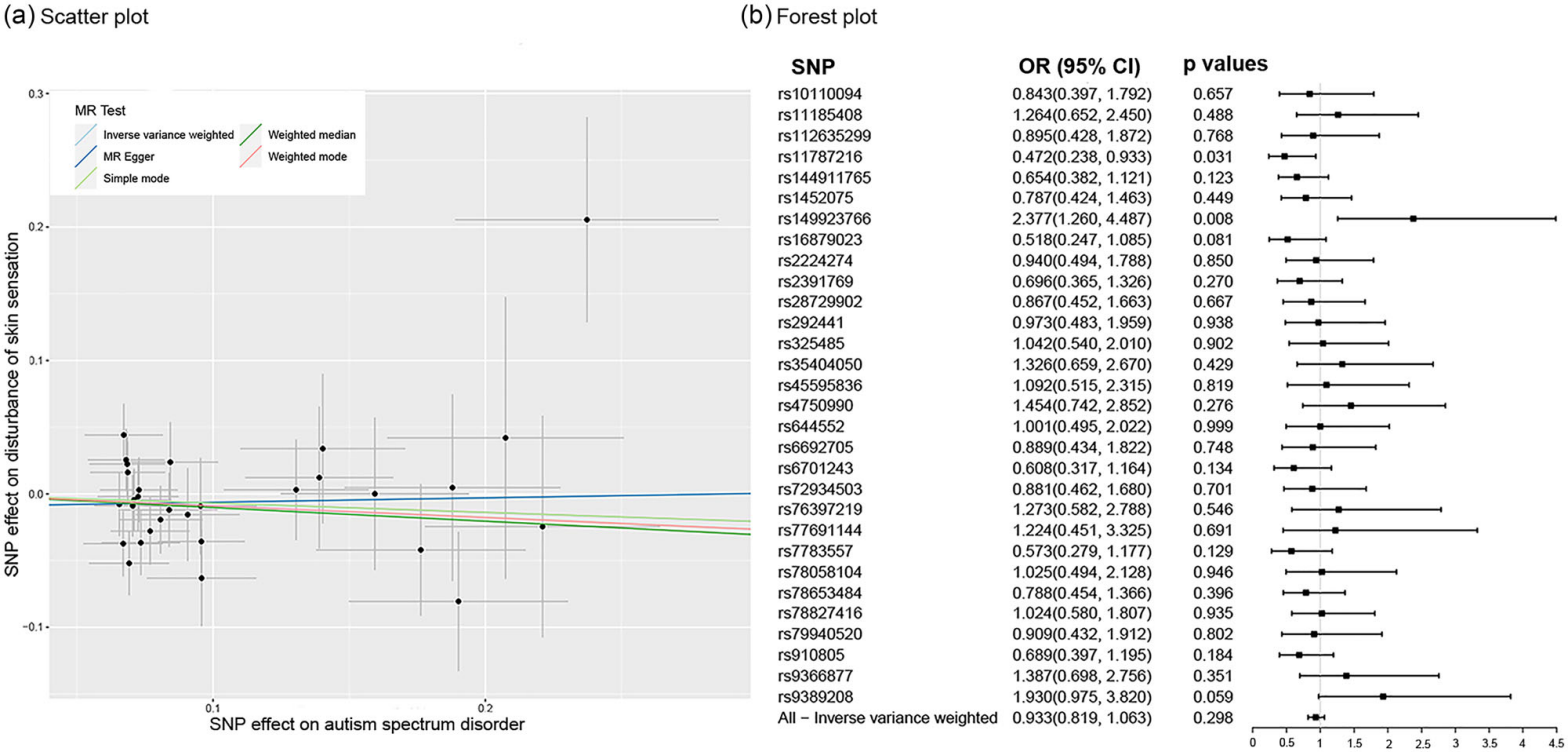
Mendel randomization diagram of causal relationship between autism spectrum disorder (ASD) and disturbance of skin sensation (DSS). A diagram illustrating the breakdown of (a) the total effect of ASD on DSS. (b) Forest plot of individual and combined single‐nucleotide polymorphism (SNP) Mendelian randomization (MR)‐estimated effects sizes.

There was no significant causal effect of ASD on DSS (all *p* values were more than .05; see Figure 3). The results were consistent across sensitivity analyses (MR‐Egger: Cochran's *Q* = 32.641, *p* = .249; IVW: Cochran's *Q* = 32.934, *p* = .280; see Supporting Information Appendix 3), none of the MR‐Egger intercepts significantly deviated from zero (MR‐Egger intercept = −.009, *p* = .620; see Supporting Information Appendix 4), and the MR‐PRESSO global tests were all statistically insignificant (*p* = .383; see Supporting Information Appendix 5). That is, ASD does not have a potential causal effect on DSS, but DSS does have a potential causal effect on ASD.

## 4 DISCUSSION

Although the link between ASD and DSS is well known, the jury is still out on whether DSS causes ASD or whether ASD has an effect on DSS (Schaffler et al., 2019). This MR study suggests that DSS may have a causal effect on ASD, whereas the reverse MR analysis showed no significant evidence for a causal effect of ASD on DSS. Thus, the results of this study provide insight into the causal effect of DSS on ASD.

Previous studies have long found an association between abnormalities in skin sensations such as touch, pain, and temperature, and the core symptoms of autism (Mikkelsen et al., 2018). However, our results provide further evidence that DSS may be a potential cause of the onset or progression of core symptoms in certain patients with ASD. This is partially consistent with the observation from prospective epidemiological studies that skin sensory abnormalities (e.g., tactile and pain hypersensitivity, and higher amplitude discrimination and temporal order judgment thresholds) may be associated with social and behavioral difficulties observed in ASD (Asmika et al., 2018; Espenhahn et al., 2023; Tavassoli et al., 2016). Thus, our results suggest that skin sensitivity may represent a marker of ASD. Specifically, we used reverse MR to establish whether there is a causal effect of ASD on DSS and did not observe evidence of such effect. This result excludes the possibility of bidirectional causality between ASD and DSS and further validates our previous results.

Our findings are plausible because, according to the hypothesis of sensory prioritization (Cermak & Daunhauer, 1997), atypical sensory processing during early development blocks the typically developing children of social cognition during feedforward (Nelson, 2007; Robertson & Baron‐Cohen, 2017). A child who is frustrated with the input of dynamic sensory information may also have difficulty constructing social information into meaningful representations or, alternatively, may find social information confusing and, therefore, choose to stay away from direct exposure or exposure to social information (Robertson & Baron‐Cohen, 2017), thereby inducing the symptoms associated with ASD.

Additionally, using the leave‐one‐out analysis, we found that the variants of rs77724093 and rs117387967 may be critical for DSS to affect the pathogenesis of ASD. According to the query on the dbSNP database, rs77724093 is located on the human genome at 127365906 on chromosome 4, and rs117387967 is located at 95353350 on chromosome 7. Researchers have conducted a series of association and linkage analyses on the association between chromosome 4 and autism (Field et al., 2013). For example, Zuo et al. (2013) found that the ADH gene on chromosome 4 is a risk factor for schizophrenia in African Americans and autism in European Americans. Furthermore, some studies have reported the results of 87 sibling pairs with autism, finding a linkage between autism and chromosome 7 (Rylaarsdam & Guemez‐Gamboa, 2019). The results of this study were further confirmed by the above‐related studies. However, it must be acknowledged that the etiology of ASD is extremely heterogeneous (Jones & Lord, 2013). The various biomarkers seen in ASD research lack replication (Parellada et al., 2023). It remains to be seen whether our initial findings can be robustly replicated in subsequent studies, or whether some of the methodological difficulties can best be overcome by combining multiple studies or designing large‐scale studies with a consistent methodology.

Currently, there is no effective intervention for ASD worldwide (Sharma et al., 2018); therefore, the findings that DSS is a biomarker in some patients with ASD can provide essential insights into the early identification and treatment of ASD. First, we propose that skin sensory sensitivity can be used as an objective indicator for early identification of ASD. By observing atypical characteristics in early skin sensory sensitivity of infants and children, we may identify children at potential risk for developing ASD. Second, since skin sensitivity may represent a marker by which some people can be subtyped, it can help explore specific subgroups of patients who are more likely to benefit from treatment. Finally, our results also suggest that skin sensitization can be used as an intervention to alleviate the core symptoms of ASD in particular children. Some investigators have already been conducting trials on such interventions, such as early touch (Cullen‐Powell et al., 2005), dance (Chen et al., 2022), and early environmental enrichment (Derguy et al., 2018).

Nevertheless, there are some limitations in this study. First, it must be acknowledged that MR studies can only provide statistical evidence for causal associations between risk factors and outcomes (Haycock et al., 2016); thus, true causal associations need to be explored in the future in the context of the biological mechanisms of ASD and clinical findings. Second, the samples used in this study were Europeans, and it is doubtful whether our results can be extrapolated to other populations (such as Asians) because of the heterogeneity of the association between genetic variation and phenotypic traits in different ethnic or national subgroups (Smith & Hemani, 2014). Additionally, sensory symptoms have always been known to differ in patients with ASD based on various underlying characteristics (e.g., sex, age, and cognitive level) which could have influenced our results (Burgess et al., 2020). However, database and methodological limitations prevented the influence of these factors from being excluded in this study; therefore, the generalization of our results should be treated with caution.

## 5 CONCLUSIONS

Although previous studies have found that skin sensory abnormalities are often present in patients with ASD, the causal relationship between these abnormalities and the core symptoms of ASD remains unknown. In the absence of a randomized controlled trial, we used a bidirectional MR study to explore the potential causal relationship. We found that DSS may be a potential cause of the onset or exacerbation of core symptoms in some patients with ASD. The discovery of DSS as a biomarker in some patients with ASD provides an objective and quantifiable indicator of the clinically relevant processes in ASD and helps in the selection of intervention targets or therapeutic approaches.

## AUTHOR CONTRIBUTIONS

**Xiao Zhong**: Conceptualization; formal analysis; investigation; methodology; software; validation; writing—original draft. **Letong Wang**: Investigation; methodology; project administration; writing—review and editing. **Lin Xu**: Investigation; software; writing—review and editing. **Jie Lian**: Investigation; software; validation; writing—review and editing. **Jie Chen**: Investigation; validation; writing—review and editing. **Xinxin Gong**: Investigation; validation; writing—review and editing. **Yongcong Shao**: Conceptualization; investigation; methodology; project administration; writing—review and editing.

## CONFLICT OF INTEREST STATEMENT

The authors declare that this research was conducted in the absence of any commercial or financial relationships that could be construed as a potential conflict of interest.

## FUNDING INFORMATION

No funding was received for this research.

### PEER REVIEW

The peer review history for this article is available at https://publons.com/publon/10.1002/brb3.3238.

## Supporting information

Appendix
**Appendix 1 Basic information on SNP loci**.
Appendix 2 Results of MR analysis.
Appendix 3 Results of heterogeneity analysis.
Appendix 4 Results of pleiotropy analysis.
Appendix 5 Results of MR‐PRESSO analysis.
